# Neuronal Encoding of Self and Others’ Head Rotation in the Macaque Dorsal Prefrontal Cortex

**DOI:** 10.1038/s41598-017-08936-5

**Published:** 2017-08-17

**Authors:** M. Lanzilotto, M. Gerbella, V. Perciavalle, C. Lucchetti

**Affiliations:** 1 Department of Medicine and Surgery, University of Parma, Parma, 43125 Italy; 2 Istituto Italiano di Tecnologia (IIT), Center for Biomolecular Nanotechnologies, Lecce, and Brain Center for Social and Motor Cognition, Parma, 73010 Italy; 3 Department of Biomedical and Biotechnological Sciences, University of Catania, Catania, 95125 Italy; 4 Department of Biomedical Sciences, Metabolic and Neuroscience, Section of Physiology and Neuroscience, University of Modena and Reggio Emilia, Modena, 41125 Italy; 5 Interdepartment Facilities Center, Section of Polyclinic, University of Modena and Reggio Emilia, Modena, I-41125 Italy

## Abstract

Following gaze is a crucial skill, in primates, for understanding *where* and *at what* others are looking, and often requires head rotation. The neural basis underlying head rotation are deemed to overlap with the parieto-frontal attention/gaze-shift network. Here, we show that a set of neurons in monkey’s Brodmann area 9/46dr (BA 9/46dr), which is involved in orienting processes and joint attention, becomes active during self head rotation and that the activity of these neurons cannot be accounted for by saccade-related activity (*head-rotation neurons*). Another set of BA 9/46dr neurons encodes head rotation performed by an observed agent facing the monkey (*visually triggered neurons*). Among these latter neurons, almost half exhibit the intriguing property of encoding both execution and observation of head rotation (*mirror-like neurons*). Finally, by means of neuronal tracing techniques, we showed that BA 9/46dr takes part into two distinct networks: a dorso/mesial network, playing a role in spatial head/gaze orientation, and a ventrolateral network, likely involved in processing social stimuli and mirroring others’ head. The overall results of this study provide a new, comprehensive picture of the role of BA 9/46dr in encoding self and others’ head rotation, likely playing a role in head-following behaviors.

## Introduction

In primates, shifting attention in space is essential for perceiving stimuli from different sensory modalities and selecting the most appropriate behavioral response^1^. Usually, an overt shift of attention simply requires eye movement, but when stimuli fall on the edge of or outside the visual field, head rotation becomes essential for orienting to those stimuli^2^. This frequently occurs during gaze following, an ability shared by humans and monkeys^3^, which often requires head rotation in order for the primate to identify *where* and *at what* others are looking.

The neuronal substrates of head-rotation control are deemed to overlap with the eye-movement network, which has been extensively studied^4^. The prevailing view indicates that a network of parietal and frontal areas forms an attention- and gaze-control network^5, 6^. Interestingly, within this network, the lateral intraparietal area (LIP) has been shown to host neurons with the capacity to “mirror the attention of others”^7^, suggesting that “gaze following behavior is mediated by a relatively straightforward system beginning with the superior temporal sulcus (STS) and proceeding directly to the attention- and gaze-control network”^3, 7^. However, when larger, overt shifts of attention are needed, eye movements are insufficient and head rotation becomes essential. Nonetheless, the current knowledge of the neurophysiological mechanisms underlying head rotation is limited to the principal ocular regions^8–13^, and the neuronal substrates of head following remain largely unknown.

The portion of the dorsal prefrontal cortex formed by Brodmann areas 9 and 46dr (BA 9/46dr) is a plausible candidate for mediating head rotation in both individual and social settings. Indeed, anatomical studies have shown that BA 9/46dr is strongly linked with eye-related areas^14–16^, and intracortical microstimulation studies have assigned to it an important role in eye and ear motor control^17^. Furthermore, transcranial magnetic stimulation applied to a large portion of the monkey’s dorsal prefrontal cortex has been shown to elicit head-rotation movements^18^. In line with these findings, human studies have suggested that the dorsomedial prefrontal cortex plays a role in joint attention^19–21^, whereas in monkeys it has been identified as a node of the face network^22^. Nevertheless, no studies have investigated the single-neuron correlates of execution and observation of head rotation, likely because of the considerable technical difficulties associated with such research. Indeed, neural recording during self head rotation is a challenging task because of mechanical artifacts, signal instability caused by head movement, and difficulties in controlling eye movement during head rotation.

Here, in order to overcome these problems, we recorded the activity of single neurons in BA 9/46dr with a dedicated setup consisting of both a multipurpose robot (MUPRO)^23^, which enabled us to monitor active head rotation as well as the forces applied by the monkey’s head during different experimental conditions (execution and observation) in both head-restrained and partially unrestrained conditions, and a search-coil system, which allowed us to simultaneously monitor eye movements.

We found that a set of BA 9/46dr neurons became active during self head rotation. Another set of neurons were visually triggered by the observed head rotation performed by another agent; among these neurons, almost half exhibited the intriguing property of enhancing their activity during both execution and observation of head rotation. In order to shed light on the possible anatomical network underlying self and others’ head-rotation processing and to better interpret functional results, we carried out a neural-tracing study on two naïve monkeys, targeting the same sector of BA 9/46dr explored in the neurophysiological study. We identified two main anatomo-functional networks that link BA 9/46dr both to a dorso/mesial fronto-parietal network, which may play a role in orienting head/gaze in space, and to a lateral temporo-prefrontal network, which may be involved in processing social stimuli, such as faces, and in mirroring others’ head rotation. The overall results of this study suggest a role of BA 9/46dr in encoding self and others’ head rotation, thus playing a possible role in head-following behaviors.

## Results

**Figure 1:**
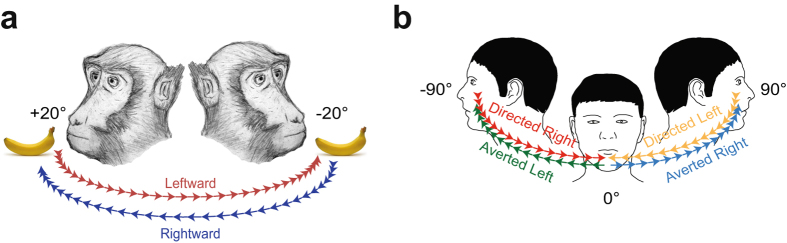
Behavioral tasks. (**a**) Head-rotation task (HRe). Through the MUPRO system (see Materials and Methods for more details), the monkey’s head was partially unrestrained. Monkey’s cartoon was drawn by author and previously used in ref. 76. (**b**) Head-rotation observation task (HRo). The monkey’s head was restricted in the central position during this task (i.e., forces were measured through the MUPRO system, but head rotation was not allowed). The experimenter, staring at the monkey (0°), turned his head with two fixed sequences: first, from 0° to 90° rightward relative to the monkey’s face (*Averted Right*, blue) and then, with no relevant delay, back from 90° to 0° toward the monkey’s face (*Directed Left*, yellow); second, from 0° to 90° leftward (*Averted Left*, green) and then, with no relevant delay, back from 90° to 0° toward the monkey’s face (*Directed Right*, red). Monkeys were randomly rewarded, with no temporal relation to the end of trials.

Single neurons in BA 9/46dr were tested during the execution of a head-rotation task (HRe) consisting of turning the head and reaching 20° rightward or leftward to receive a piece of fruit as a reward (Fig. 1a). The MUPRO system (see Materials and Methods) allowed us to record active head rotation as well as the forces applied by the monkey’s head on the horizontal plane.

We recorded a total of 120 neurons from the left hemisphere of two macaques (MK1 and MK2) during the HRe. Moreover, to verify whether some of these neurons were modulated by the observation of another agent’s head rotation, 33 neurons were also tested with a head-rotation observation task (HRo) (Fig. 1b). Finally, in order to verify whether neuronal responses could be accounted for by saccade-related activity, all neurons were tested in a visually guided saccade task (ST) (Supplementary Fig. S1a). The average speed of the monkeys’ head rotation (19.88 ± 0.35°/s) was lower than that of the experimenter (28.13 ± 0.62°/s) because monkey’s head was partially anchored to the MUPRO, which imposed an inertial load to head rotation.

Among the recorded neurons, 71 were considered task related: 44 discharged during the HRe (*head-rotation neurons*) and 27 during the HRo (*visually triggered neurons*), and about half of these latter neurons (n = 12) discharged during both the HRe and HRo (*mirror-like neurons*).

**Figure 2:**
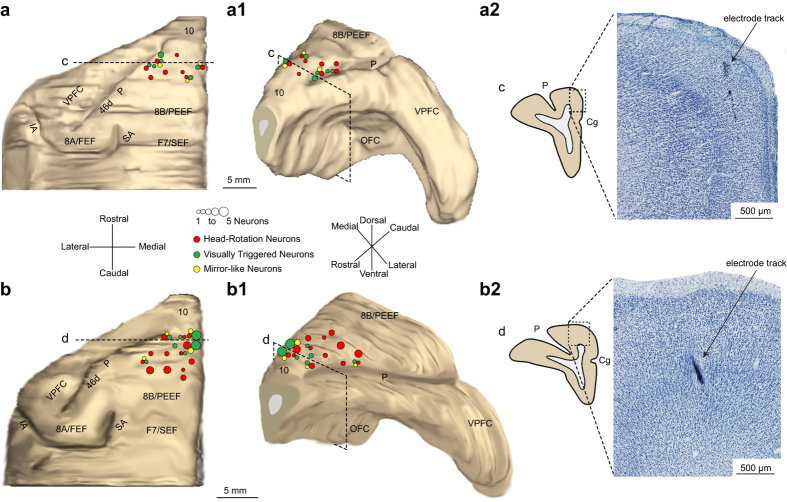
Histological reconstruction of the recorded regions and functional maps of MK1 and MK2. (**a**,**b**) 3-D reconstruction (dorsal view) of the left hemisphere of MK1 (**a**) and MK2 (**b**), with superimposed number and class of neurons recorded from each site. (**a1**,**b1**) 3-D reconstruction (rostro-lateral view) of the same hemispheres of MK1 (**a1**) and MK2 (**b1**). (**a2**–**b2**) Examples of coronal sections at high magnification (photomicrograph of Nissl-stained section), showing where electrode tracks were identified. Dashed black lines in (**a**,**b**) and dashed black boxes in (**a1**–**b1**), labeled “c” and “d” indicate the position from which each coronal section was taken. SA, superior arcuate sulcus; IA, inferior arcuate sulcus; P, principalis sulcus; VPFC, ventral prefrontal cortex; OFC, orbitofrontal cortex.

The anatomical location of the recorded neurons has been confirmed histologically (see Supplementary Information). Figure 2 shows the reconstruction of the spatial distribution of the recorded neurons in both animals superimposed on a 3-D reconstruction of each monkey’s brain (Fig. 2).

### Head-rotation neurons

**Figure 3:**
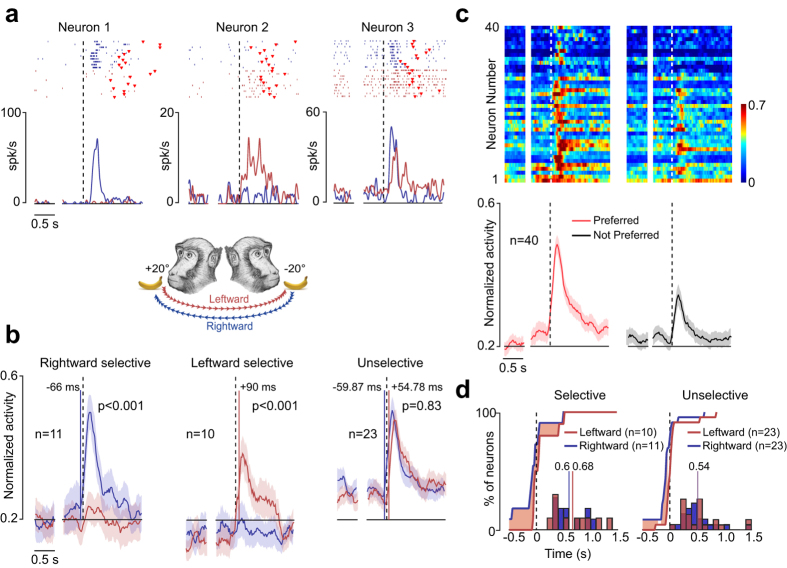
Functional properties of BA 9/46dr head-rotation neurons. (**a**) Examples of three head-rotation neurons. For each neuron, rasters and spike-density function on the left exemplify the baseline activity. After the gap, the activity is aligned (dashed lines) to the head movement onset, and the period immediately before the dashed line represents the neural activity during the premovement epoch. Red markers represent the movement offset. (**b**) Population activity of three different types of head-rotation neurons (Rightward selective, Leftward selective and Unselective). Population activity is aligned as single-neuron examples. The colored shaded area around each curve represents 1 standard error. The colored lines superimposed on the population activity represent the time of population-activity onset. *Rightward selective* (F = 15.72, p < 0.001) and *Leftward selective* (F = 12.11, p < 0.001) neurons exhibited directional selectivity during the head-rotation phase, whereas *Unselective* neurons did not exhibit any directional tuning (F = 0.19, p = 0.83). (**c**) Population plot of excitatory head-rotation neurons aligned (dashed lines) as in (**b**) and grouped based on their directional preference. In the top part, each row represents a single neuron, whereas the bottom part shows the averaged population plot. Alignments and conventions are the same as in panel (a). (**d**) Curves represent cumulative distribution of neurons’ latencies relative to the movement onset of selective and unselective head-rotation neurons during rightward (blue) and leftward (red) head rotation. The red shaded areas represent the time interval in which the proportion of neurons during rightward (blue) and the proportion during leftward (red) head rotation were significantly different from each other (χ^2^ test performed bin per bin, bin = 100 ms, p < 0.05). Histograms describe the distribution of burst duration for selective (left plot) and unselective (right plot) head-rotation neurons during rightward (red) and leftward (blue) head rotation. The colored lines superimposed on the histograms represent the averaged burst duration.

Figure 3a shows examples of head-rotation neurons (n = 44). Almost half of them (n = 21, 48%) exhibited a directional selectivity either for rightward (Neuron 1) or leftward (Neuron 2) head rotation, whereas the remaining instances (n = 23, 52%) did not exhibit any significant directional preference (Neuron 3). Figure 3b shows that for these three sets of neurons, the population response and directional tuning are as robust as they are at the single-neuron level. Figure 3c shows the response profile of all the excitatory neurons (n = 40) based on the direction for which they exhibited the absolutely strongest (preferred) or weakest (not preferred) discharge, emphasizing the directional tuning of the entire neuronal population. It is important to note that rightward (n = 21) and leftward (n = 19) directional preferences were similarly represented (χ^2^ = 0.1, p = 0.75).

Interestingly, our comparison of single-neuron firing properties (Fig. 3d) highlights the presence of a faster (63 ms before head-rotation onset) onset during rightward (contralateral) head rotation relative to leftward (ipsilateral) head rotation (72 ms after head-rotation onset). This effect was significant even when we separately tested the two subpopulations, that is, selective and unselective neurons. Finally, rightward and leftward head rotation were encoded by bursts of similar duration for both selective (t = −0.55, p = 0.59) and unselective (t = 0.02, p = 0.98) neurons (Fig. 3d, histograms).

Because it is known that gaze shifts greater than 20° usually require head rotation^2^, it could be argued that neuronal discharge during head rotation may in fact be due to a simultaneous gaze shift. Formal tests of single-neuron activity with the ST (see Materials and Methods) show that there is no significant saccade-related modulation, which is also clearly evidenced by population activity (Supplementary Fig. S1). These findings support the claim that BA 9/46dr neurons do encode self head rotation.

### Visually triggered neurons

**Figure 4:**
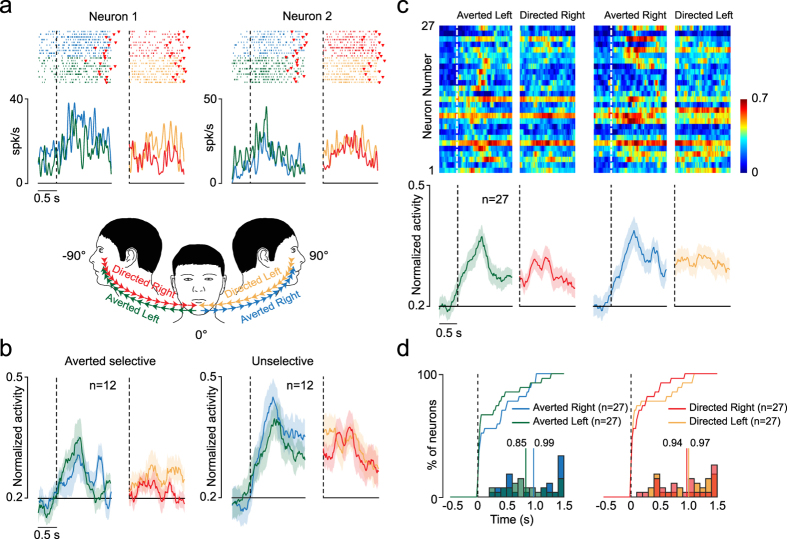
Functional properties of visually triggered neurons. (**a**) Examples of two visually triggered neurons exhibiting different selectivity for the direction of the experimenter’s head rotation. For each neuron, rasters and spike-density function are aligned (dashed lines) to the onset of the experimenter’s head rotation (see schematic drawing below neuron examples), averted (green and light blue) or directed (red and orange, after the gap). The time epoch before the first dashed line is used as the baseline, in which the experimenter faced the monkey and remained still. Red markers represent the movement offset. (**b**) Population activity of visually triggered neurons aligned as the single-neuron examples. The colored shaded area around each curve represents 1 standard error. (**c**) Normalized activity of visually triggered neurons aligned (white dashed lines) to the experimenter’s head-movement onset. Each row represents a single neuron. The average population activity is shown on the bottom of each panel. Other conventions as above. (**d**) Curves represent the cumulative distribution of visually triggered neurons’ latencies relative to the onset of the experimenter’s head rotation in different directions (color code). In an χ^2^ test performed bin per bin (bin = 100 ms) between cumulative distributions, we did not observe significant differences in the proportion of neurons (χ^2^, p > 0.05). Histograms on the bottom show the distribution of burst durations for all visually triggered neurons during the experimenter’s head rotation in different directions (color code as before). The colored bars and associated values specify the average burst duration in each condition.

Most of the neurons tested during the HRo were visually triggered (27/33, 82%), increasing their activity during at least one of the four tested epochs (see Materials and Methods for details and Fig. 1). Figure 4a shows examples of visually triggered neurons. About half of them (n = 12, 44.5%) discharged only during *Averted* epochs (Neuron 1), with a preference for leftward rotation in terms of the number of single neurons (n = 7 *Averted Left* selective; n = 5 *Averted Left* and *Right* selective; n = 0 *Averted Right* selective, χ^2^ = 6.5, p = 0.038). The remaining 12 visually triggered neurons (44.5%) did not exhibit any selectivity for the direction of head rotation (Neuron 2). Only 3 neurons (11%) were selectively activated during *Directed* epochs. Figure 4b shows the directional tuning of selective neurons, which is significant also at the population level (F = 7.10, p = 0.02); this is not the case for unselective neurons (F = 3.44, p = 0.09). Figure 4c shows the response profile of all visually triggered neurons (n = 27) during all epochs of the HRo. Although observing the experimenter’s averting of the head from the monkey appeared to be the most effective stimulus, this difference did not reach statistical significance (F = 1.79, p = 0.19). Likewise, absolute movement direction was similarly represented at the population level (right vs. left, F = 0.07, p = 0.79; Fig. 4c). Interestingly, our comparison of single-neuron firing properties (Fig. 4d) highlighted that about half of the visually triggered neurons exhibited a fast discharge onset (within the first 100 ms from the beginning of the experimenter’s head rotation), and no significant difference was found between directions of rotation (Fig. 4c, cumulative distributions). Finally, the experimenter’s head rotation was encoded by bursts of similar duration for both Averted (t = 1.22, p = 0.22) and Directed (t = 0.23, p = 0.82) epochs (Fig. 4d, histograms).

Because during the HRo the monkey’s head was restricted and it was not required to maintain fixation during the experimenter’s head rotation, the application of isometric neck forces or different types of eye-movement patterns could produce the neural response recorded during the observation of head rotation. Indeed, we observed that during the HRo, monkeys produced mainly smooth-pursuit eye movements, but in some trials, saccades were also observed (Supplementary Fig. S2a). However, our control experiment with the ST enabled us to exclude saccadic eye movements as a cause of visually triggered neuronal activity (Supplementary Fig. S2b). In addition, by comparing visually triggered neuronal activity between trials characterized by saccades or smooth pursuits (Supplementary Fig. S2c), we were also able to exclude different types of eye movement as a cause of the discharge. Finally, by measuring the isometric neck forces applied on the horizontal plane by monkeys while recording visually triggered neurons (Supplementary Fig. S3a and b), we were able to exclude the monkey’s attempt to rotate the head as an explanation for the observed discharge. Therefore, the present findings constitute the first evidence of BA 9/46dr neurons triggered by the observation of others’ head rotation.

### Mirror-like neurons

**Figure 5:**
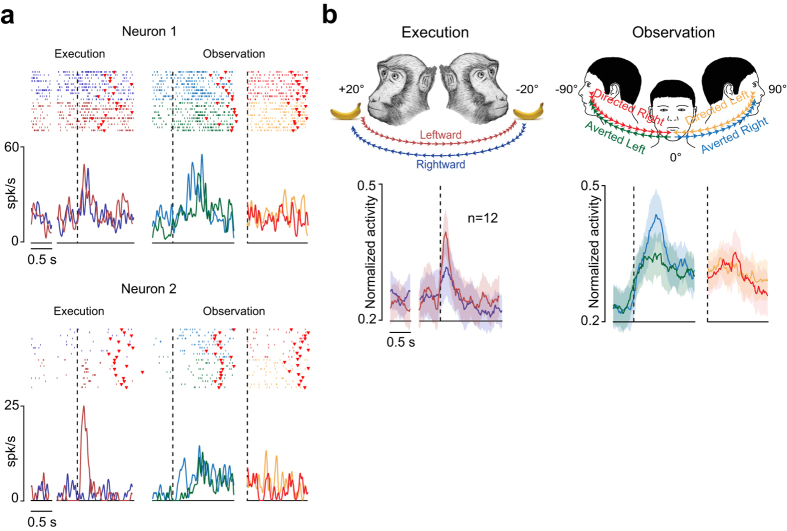
Functional properties of BA 9/46dr mirror-like neurons. (**a**) Examples of two mirror-like neurons exhibiting enhancement of their activity during both the HRe (left) and HRo (right). For each neuron, rasters and spike-density function are aligned (dashed lines) to the monkey’s (left plot) and experimenter’s (right plots) head-rotation onset. Red markers represent the movement offset. Conventions as in Figs 3 and 4. (**b**) Upper part, schematic representation of the HRe and HRo, indicating the color codes used for the population plots below. Bottom part, population activity of mirror-like neurons. Population activity is aligned as single-neuron examples. The colored shaded area around each curve represents 1 standard error. Other conventions as in Figs 3 and 4.

One of the most interesting findings of this study is that almost half of the visually triggered neurons (12/27, 44%) that became active during observation of head rotation also discharged when the monkey rotated its head, thus exhibiting mirror-like behavior. Figure 5a shows examples of mirror-like neurons. Neuron 1 responded during the HRe with no directional selectivity, but also exhibited enhanced activity during the HRo, particularly during *Averted* epochs. Neuron 2 is another example of a mirror-like neuron that exhibited a selective response for the monkey’s leftward head rotation in the HRe, whereas it responded with no directional selectivity during the HRo. Figure 5b shows the population activity of all the mirror-like neurons: a significant response is present during both execution (F = 4.59, p = 0.021) and observation (F = 21.57, p < 0.001) of head rotation, in line with the results of single-neuron analysis. Details about the directional preferences of mirror-like neurons are shown in Table 1. Interestingly, about half of the mirror-like neurons (n = 7, 58%) exhibited directional tuning during self head rotation, whereas directional tuning during the observation of head rotation was less frequently observed (n = 3, 25%).

**Table 1:** Table shows details about selective and/or unselective responses of mirror-like neurons.

Obs	Averted Selective		Averted Unselective	Directed Selective		Unselective	Tot
	Right	Left		Right	Left		
Exe							
Rightward Selective	0	0	0	0	1	2	3
Leftward Selective	0	2	0	0	0	2	4
Unselective	0	0	2	0	0	3	5
Tot	0	2	2	0	1	7	12

These findings represent the first evidence of the existence of neurons with mirror-like properties related to head rotation, and they suggest that in addition to neuronal populations encoding self and others’ head rotation, BA 9/46dr hosts neurons encoding head rotation in an agent-independent manner.

### Anatomical connections of BA 9/46dr

In order to explore the possible anatomical network underlying self and others’ head-rotation processing, we injected neural tracers (retro-anterograde Lucifer Yellow Dextran, LYD, and retrograde Wheat Germ Agglutinin-horseradish, WGA) into two naïve monkeys, MK3 and MK4, targeting the same sectors of BA 9/46dr investigated in the electrophysiological study carried out in MK1 and MK2 (see Supplementary Information). To this purpose, the functional maps of MK1 and MK2 were overlapped on the anatomical reconstruction of their brains (see Fig. 2) and then superimposed on MRI scans of the brains of MK3 and MK4.

Considering the overall pattern of anatomical connections identified in this study, we suggest that two main anatomo-functional networks link the region in which head-rotation neurons were found with a dorso/mesial and a lateral set of brain areas: the dorso/meZZsial network includes mesial/parietal and dorsolateral frontal areas that may be involved in head/gaze shift in space, whereas the lateral network encompasses temporal and ventrolateral prefrontal areas, likely conveying information about social stimuli such as faces and others’ head direction.

### Self dorso/mesial head/gaze-orienting network

**Figure 6:**
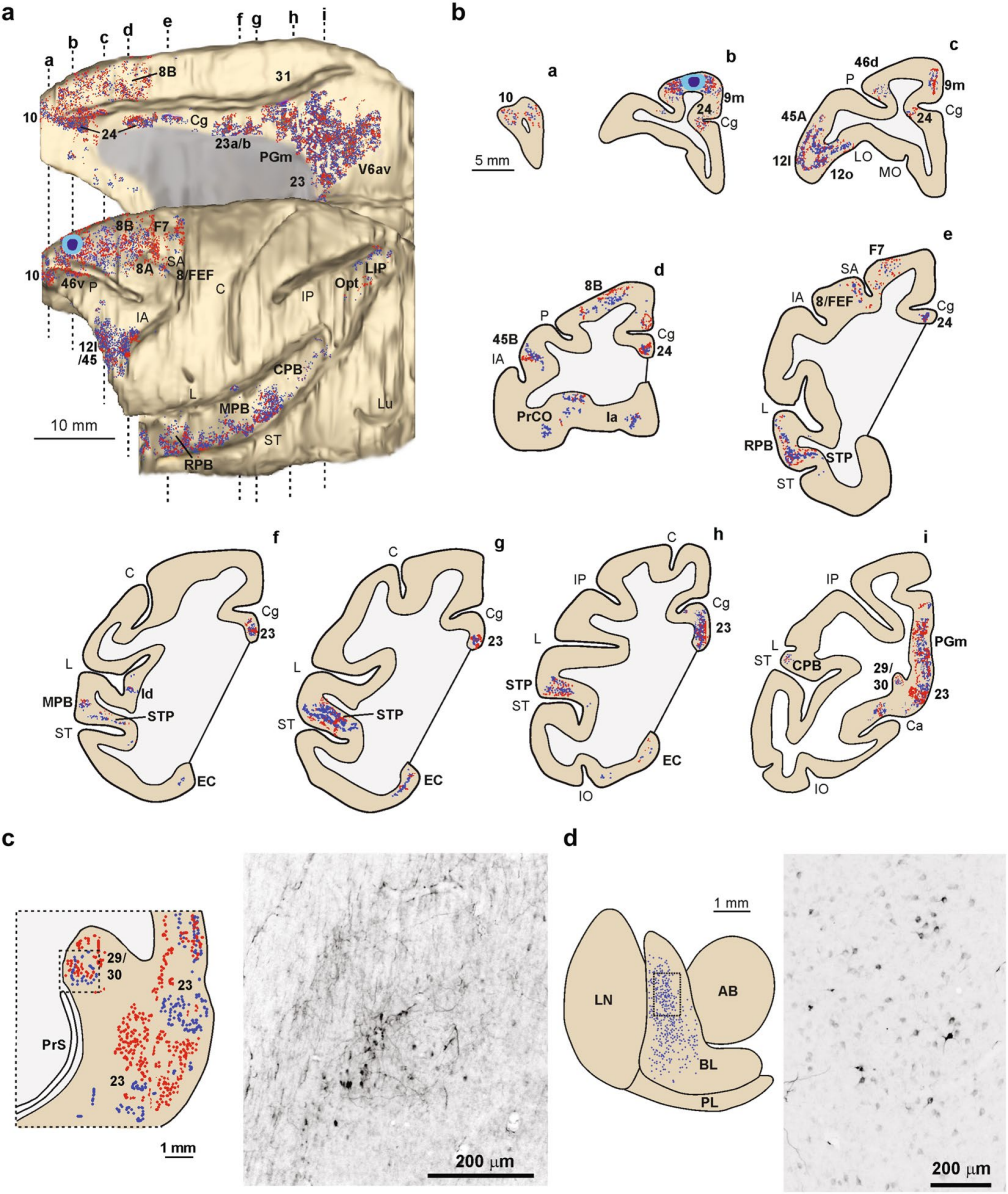
Distribution of the anterograde (red dots) and retrograde (blue dots) labeling observed following LYD injection in MK3, shown in a dorsolateral and a mesial view of the 3-D reconstruction (**a**) and in drawings of coronal sections (**b**) arranged in a rostral-to-caudal order (**a**–**i**). Each blue dot corresponds to one labeled cell, whereas each red dot corresponds to 15–25 labeled terminals. The levels at which the sections were taken are indicated by dashed lines in the dorsolateral and mesial view of the 3-D reconstructions. (**c**) Drawing, left, and photomicrograph, right, of the retrosplenial cortex. (**d**) Drawing, left, and photomicrograph, right, of the amygdala. The dashed box indicates the location of the photomicrograph. AB, accessory basal nucleus; BL, basolateral nucleus; C, central sulcus; IP, intraparietal sulcus; L, lateral fissure; Ln, lateral nucleus; Lu, lunate sulcus; PL, paralaminar nucleus, ST, superior temporal sulcus. Other abbreviations as in Fig. 2.

Strong connections of the injected BA 9/46dr region were observed in the adjacent area 10 and 8B/PEEF (Fig. 6a, sections a and d, and Supplementary Fig. S4a, sections a and d), involved in decision-making^24^ and in the control of ear/eye movements for detecting auditory stimuli in space^25–31^, respectively. Strong connections were also found with the dorsal part of area 8 A/FEF, involved in controlling large saccades^32^ and head movements^33, 34^, and with the agranular area F7, in a sector compatible with supplementary eye fields (SEF; Fig. 6b, section e, and Supplementary Fig. S4a, sections e and f), which play a role in head, in addition to eye, motor control^8, 12^. Following LYD injection in MK3, we also found labeling in oculomotor-related areas of the inferior parietal lobule, namely, LIP (caudal and dorsal part) and Opt (Fig. 6a). Concerning subcortical structures, labeled terminals involving basal ganglia were found mainly in the caudate sectors taking part in the oculomotor-related loop^35^. Finally, following LYD injection, labeled terminals were observed even in the deep layers of the superior colliculus, involved in the translation of sensory signals (visual, auditory, and somatosensory) into motor commands for the control of saccadic eye movements^36^. These findings support the claim that BA 9/46dr could be considered part of a network related to attention and head/gaze motor control. Concerning connections with mesial brain regions, dense labeling was found in the rostral-most part of the anterior cingulated cortex, especially following LYD injection (Fig. 6b, sections b and c). Additional strong connections were found with the mesial parietal and retrosplenial cortex (Fig. 6b, section i, Fig. 6c, Supplementary Fig. S4a, section m, and Supplementary Fig. S4b) and, in MK3, with the entorhinal cortex (Fig. 6b, sections g and h). Interestingly, these areas, as well as the hippocampus, play a role in navigation^37–40^, and researchers have found *head-direction cells* that discharge selectively when the animal’s head points at a specific azimuthal direction^38, 41^. Both anatomical and physiological results regarding BA 9/46dr could lead to an interesting hypothesis: this brain region may play a role in head motor control for navigation, possibly contributing to an egocentric representation of the surrounding space. Indeed, rotating the head on the horizontal plane is a fundamental requirement for building a neuronal representation of head position relative to the azimuth.

### Others’ ventrolateral head-direction network

Strong connections were observed with the ventrolateral prefrontal area 45 and adjacent area 12 l (Fig. 6b, section c, and Supplementary Fig. S4a, section d), deemed to play a crucial role in the processing of visual and auditory stimuli related to communicative behavior^42, 43^, and with the orbitofrontal area 12o, whose activation has been linked to observed face processing in previous fMRI studies^44^. Outside the frontal lobe, strong connections were found both with the temporal cortex (Fig. 6b, sections e–i, and Supplementary Fig. S4a, sections g–m), involving almost the entire extent of the parabelt regions that play a role in the processing of complex sounds, such as voices and their spatial location^45, 46^, and with the superior temporal polysensory area (STP), which hosts neurons responsive to faces, body parts, and others’ gaze direction^47, 48^. Following LYD injection, we also found labeling in the middle insular region (Fig. 6b, section f), associated with affiliative behavior, especially during gaze contact^49–51^. Concerning subcortical structures, following LYD injection labeled cells were observed in the amygdala (Fig. 6d), involved in coding face stimuli and gaze following^52^, and labeled terminals in the periaqueductal gray. These findings support the claim that BA 9/46dr may be part of a lateral network that contributes information about social stimuli such as faces and others’ head direction.

## Discussion

This study provides the first single-neuron evidence of different types of neurons related to self and/or others’ head rotation in BA 9/46dr. Although with our paradigm we could not discriminate between contribution of proprioceptive, vestibular or purely motor signal to neuronal discharge during HRe, we found neurons related to self head rotation (*head-rotation neurons*), other neurons triggered by the sight of others’ head rotation (*visually triggered neurons*), and another set discharging during both self and others’ head rotation (*mirror-like neurons*). Furthermore, connectional data suggest that these neuronal properties could derive from the convergence in BA 9/46dr of two main anatomo-functional networks: a self dorso/mesial head/gaze-orienting network and an others’ ventrolateral head-direction network. These two networks may convey the necessary information for the emergence of self, others’, and agent-independent head-direction coding.

The dorsal prefrontal cortex, including area 9/46dr, is deemed to play a role in orienting processes^17, 28, 30^, and it hosts neurons encoding both ear and eye movements, which are crucial for focusing on complex stimuli that fall into different spatial regions^25–27, 29, 31^. In line with these studies, we found that a set of BA 9/46dr neurons discharge with a strong directional tuning during voluntary head rotation and that their activity is not accounted for by saccade-related activity (Supplementary Fig. S1). This suggests that individual cortical neurons within head/gaze-related brain regions separately encode eye and head displacement, which is in line with previous evidence^10^. Even though one might expect an overrepresentation of the contralateral motor field, we found that the number of neurons with contralateral or ipsilateral directional selectivity was balanced (Fig. 3b). This finding may result from the involvement of BA 9/46dr in high-order executive functions, thus needing information coming from both spatial hemifields in order to organize complex behaviors. Nevertheless, we found a faster discharge onset during rightward (contralateral) relative to leftward (ipsilateral) head rotation (Fig. 3d), which can be explained by the presence in the prefrontal cortex of neurons with premotor-like properties^10, 53^, playing a role in planning and controlling movement of contralateral side of the body. In contrast, during head rotation toward the ipsilateral side, sensory signals, such as proprioceptive and vestibular ones as observed in other brain regions^54–58^, may allow head rotation neurons to play a role in head rotation monitoring. Although further studies are needed to clarify these issues, our findings together with the well-known role of this region in attentional processes^59^, suggest that BA 9/46dr may be part of a network involved in controlling and monitoring head shifts as normally happens in social situations during head following behavior.

However, in order to follow the shift of the head of others, it is necessary to extract information about their head direction. Interestingly, in BA 9/46dr, we also found visually triggered neurons encoding others’ head rotation (Fig. 4), despite we could not directly demonstrate that observed head rotation was the optimal stimulus that maximized the cell’s activity, since we could not test a broader range of moving stimuli. Nonetheless, *Averted* head rotation appeared to be the most effective stimulus (44.5% of visually triggered neurons preferred *Averted* head rotation, whereas only 11% were selective for *Directed* head rotation), which is in line with studies performed in other brain regions that evidenced the same bias for averted gaze shifts^52, 60^. This effect could be due to the fact that faces with an averted gaze point toward a wider space sector and a less immediately identifiable target than do those with a directed gaze^52^, hence inducing more exploration. Surprisingly, despite we recorded from both monkeys’ left hemisphere, no neuron exhibited a selective response for *Averted Right* head rotation (see Results section). A possible explanation is that a spatial remapping does occur, similar to the remapping previously demonstrated for hand actions in the human anterior parietal cortex^61^, where the observation of actions seen from an allocentric viewpoint (as in the present study) generated greater activation in the ipsilateral hemisphere.

About half of the visually triggered neurons also exhibited the additional intriguing property of *mirroring head rotation*, whereby their activity increased during both execution and observation of head rotation (*mirror-like neurons*, Fig. 5). These latter neurons behave like the classic *mirror neurons* ^62–65^ described in many other brain regions^66^ and species^67^. Accordingly, we found a lower directional tuning during the observation of head rotation than during the execution task (Table 1), which is in line with the general property of mirror neurons in different areas that exhibit a lower degree of visual relative to motor selectivity (e.g., for type of grip, direction of the stimulus, or features of the stimulus)^64, 68, 69^. Neurons with similar features, which can “mirror the attention of others”, have been reported in the parietal area LIP^7^, involved in eye-gaze control. Therefore, to our knowledge, this study provides the first evidence of monkey neurons involved in *mirroring head rotation*.

Our connectional data support our functional findings and provide an important framework for explaining the functional properties of BA 9/46dr above described (Fig. 6 and Supplementary Fig. S4). Indeed, we identified two main anatomo-functional cortical networks. The first is a dorso/mesial network, encompassing head/gaze-related brain regions such as the dorsal part of FEF^33, 34^, SEF^8, 12, 70^, PEEF^30^, and the parietal, retrosplenial, and entorhinal cortex^38, 41^. This network may be involved in the transformation of sensory signals into head motor commands in order to drive shifts of attention and rotate the head toward a specific spatial region. The second is a ventro/lateral network, including mainly temporal and ventrolateral prefrontal areas. Facial-sensitive and/or head/gaze-selective visual responses have been found in most of the areas of this network: STP^47, 48^, STS^71^, the ventral prefrontal cortex^42, 72, 73^, and the orbitofrontal cortex^44^. Interestingly, the reversible inactivation of the posterior superior temporal sulcus (pSTS) suppresses social gaze following in macaques^74^. Thus, BA 9/46dr may be an important cortical node for the integration of information needed for the emergence of self, others’, and mirror head-direction coding underlying complex behaviors, such as head following.

The overall results of this study provide a new, comprehensive picture of the role of BA 9/46dr in encoding self and others’ head rotation, likely playing a role in head following behaviors.

## Materials and Methods

### Subjects

Physiological experiments were carried out at the University of Modena and Reggio Emilia on two (MK1 and MK2) adult female macaque monkeys (*Macaca fascicularis*, 3–4 kg, 4–5 years old). Subjects’ water intake was controlled daily to enhance motivation, and they were trained to sit in a primate chair and to interact with the experimenters. Two (MK3 and MK4) naïve monkeys (*Macaca fascicularis* and *Macaca mulatta*, 3–4 kg, 4–5 years old) were used for injection of neural tracers. The anatomical experiments were carried out at the University of Parma. All experimental protocols complied with the European law on the humane care and use of laboratory animals (directives 86/609/EEC, 2003/65/CE, and 2010/63/EU), were authorized by the Italian Ministry of Health (n. 65/2010-B e n. 66/2010-C released on 03/29/2010; n. 155/2013-C released on 06/25/2013; n. 294/2012-C released on 12/11/2012; n. 48/2016-PR released on 01/20/2016), and were approved by the local Ethics Committee of the University of Modena and Reggio Emilia and University of Parma.

### Recording

Monkeys (MK1 and MK2) sat in the primate chair in front of a panel at a distance of 114 cm, on which 49 bicolored light-emitting diodes (LEDs) with a diameter of 0.05° were placed. Eye position was monitored by using a magnetic search coil surgically implanted beneath the conjunctiva of one eye and sampled at 1 KHz^75^. Head position was maintained with a surgically implanted stainless steel prosthesis, and monkeys’ heads were painlessly restricted by MUPRO^23^, a homemade multipurpose neck robot. MUPRO was designed to record both the isometric neck forces and to enable head rotation on the horizontal plane. It consists of a mechanical device, comprising a cardan joint, a potentiometer, an electromagnetic brake, and four flexion load cells (FLCs), which identify the isometric forces applied in four directions of the space (i.e., forward, backward, rightward, and leftward), plus an oleodynamic system allowing head rotation on the horizontal plane between +/− 20°. These components are assembled on a column bolted to the primate’s chair. An electrical device provides DC power for the potentiometer and the brake. To allow electrophysiological recordings, macaques were additionally implanted with a stainless-steel recording chamber (Thomas Recording) using stereotaxic coordinates. The inner diameter of the recording chamber was 19 mm, and it was vertically oriented to enable a perpendicular approach to the region of interest. All surgical procedures were conducted using an aseptic technique and under general anesthesia (Zoletil 10 mg/kg i.m.), and after each surgical intervention, treatment with antibiotics, cortisone, and analgesics was administered for up to one week. Before each session, the chamber was aseptically opened and rinsed thoroughly with sterile saline, and through a microdrive system (Mini Matrix Thomas Recording) a quartz-glass electrode (0.5–1.0 MΩ) passed through the dura. The biological signal was preamplified (PreAmplifier DPA-4), amplified, and filtered (Main Amplifier/Filter System MAF-05) to eliminate artifacts from 5 to 75 KHz and coil drivers. A SPS 8701E Waveform Discriminator System selected the amplified unit activity, which was monitored using an oscilloscope and also audio-monitored. Eye position, LED levels, unit activity, auditory markers, head forces, and head-rotation signals were sampled continuously during the experimental session at 1 KHz, stored by SuperScope II (GWI) software, and imported into Matlab for further analysis by custom scripts.

### Behavioral Tasks

Once a neuron was isolated, macaque subjects performed a *head-rotation task* (HRe) (Fig. 1a). The monkey’s head was partially unrestrained (i.e., forces were measured through the MUPRO system, and head rotation of +/− 20° was allowed), and subjects received a piece of fruit (1 × 1 cm), positioned to their right or left, as a reward. In order to reach the food, they had to turn their heads and reach 20° rightward or leftward. Head forces and position were signaled by analog signals expressed in volts. Once subjects performed the HRe, neurons were also tested during a *visually guided saccade task* (ST) (Supplementary Fig. S1a) in which they first fixated, for a fixed period of 1 s, a central red LED (red period) within an electronic window ranging from 3 to 5 deg. When the central red target was abruptly switched off, a peripheral red target (20° up, down, left, or right) simultaneously appeared. Subjects shifted their gaze from fixation to this peripheral target as quickly as possible (within 0.7 s), maintaining a new fixation for 1 s to receive a juice reward. A 2-s intertrial period followed each trial. The visual stimuli were presented by homemade software running on a personal computer, and an acoustic cue, with an intensity of 40–50 dB, was switched on at the beginning of each trial session and switched off at the end, thus signaling to the monkey the beginning and the end of the working period. The onset and offset of each epoch in ST was signaled by analog LED levels. Finally, a subset of neurons recorded in BA 9/46dr was also tested during the *head-rotation observation task* (HRo; Fig. 1b). The monkey’s head was restricted in the central position (i.e., forces were measured through the MUPRO system, but head rotation was not allowed), and the experimenter, facing the monkey at a distance of 60 cm, turned his head with two fixed sequences: first, rightward (with respect to the monkey), from 0° to 90° away from the monkey’s face (*Averted Right* epoch) and then, with no relevant delay, leftward, from 90° to 0° toward the monkey’s face (*Directed Left* epoch); second, leftward, from 0° to 90° away from the monkey’s face (*Averted Left* epoch) and then, with no relevant delay, rightward, from 90° to 0° toward the monkey’s face (*Directed Right* epoch). The intertrial period between two sequences during HRo was at least of 2 s and it was used as baseline. The onset and offset of HRo epochs were signaled by analog auditory markers produced by experimenters. During the HRo, monkeys were randomly rewarded, with no temporal relation to the end of trials. Eye and head position as well as neck forces were continuously recorded during the HRe, ST, and HRo. The activity of each neuron was recorded in at least 10 trials for each basic condition. Neurons were classified as task related if they had a significant response (Bonferroni post hoc test, p < 0.05) to the HRe and/or the HRo.

### Analysis

Single-neuron activity was analyzed in relation to the analog signals related to the main behavioral events. Spikes were recorded continuously and were convolved with a 20-ms Gaussian smoothing window. Self-initiated head rotation was analyzed, synchronizing neural responses to the onset of head-rotation movements considering the following epochs: (1) baseline, from 1.5 to 1 s before head-rotation onset, during the intertrial period; (2) premovement, 0.5 s before head-rotation onset; (3) movement, 1.5 s after head-rotation onset. Possible responses to self head rotation relative to baseline, expressed as mean firing rate (spikes/s), were assessed considering both directions (rightward and leftward) by means of a 2 × 3 repeated measures ANOVA (factors: Direction, Epoch) with a significance criterion of p < 0.05. Only neurons exhibiting at least a significant effect of the factor Epoch, alone or in interaction with the other factors, were classified as head-rotation neurons (Bonferroni post hoc test, p < 0.05).

A subset of single neurons was tested during the HRo. The neuronal responses were analyzed, synchronizing the neuronal activity to the onset of experimenter’s head rotation considering the following epochs: (1) baseline, 0.5 s before movement onset, during the intertrial period; (2) averted epochs (*Averted Right*, *Averted Left*), from movement onset to movement offset (of variable duration, calculated on a trial-by-trial basis); (3) directed epochs (*Directed Left*, *Directed Right*), 1.5 s after offset of averted epochs. Possible responses to the experimenter’s head rotations relative to baseline, expressed as mean firing rate (spikes/s), were assessed considering both hemifields (right hemifield and left hemifield) by means of a 2 × 3 repeated measures ANOVA (factors: Hemifield, Epoch) with a significance criterion of p < 0.05. Only neurons exhibiting at least a significant effect of the factor Epoch, alone or in interaction with the other factors, were classified as visually triggered (Bonferroni post hoc test, p < 0.05).

Finally, saccadic eye movements were analyzed, synchronizing neural responses to the onset and offset of saccades produced during ST considering the following epochs of interest: (1) baseline, from 0.7 to 0.9 s after the red central target onset, while monkeys were fixating in central position; (2) premovement, 0.2 s before the saccade onset; (3) movement, from saccade onset to saccade offset (of variable duration, calculated on a trial-by-trial basis); (4) reaching position, 0.2 s after saccade offset. Eye onset and offset were defined as the last points on either side of the peak velocity before which the tangential velocity fell below 30°/s^17, 30^. Possible responses to the saccadic eye movement relative to baseline, expressed as mean firing rate (spikes/s), were assessed considering all directions (20° up, down, left, right) by means of a 4 × 4 repeated measures ANOVA (factors: Direction, Epoch) with a significance criterion of p < 0.05. Only neurons exhibiting at least a significant effect of the factor Epoch, alone or in interaction with the other factors, were classified as eye-motor neurons (Bonferroni post hoc test, p < 0.05).

Population analyses were carried out, taking into account single-neuron responses expressed in terms of normalized mean activity using a moving window of 200-ms slit forward in steps of 20 ms and analyzed with different repeated measures ANOVAs depending on the conditions to be compared, as previously described for single neurons. Population analyses for Fig. 4b and c were carried out by means of 2 × 2 repeated measures ANOVA (factors: Direction, Epoch) with a significance criterion of p < 0.05, followed by a Bonferroni post hoc test correction (p < 0.05). Finally, single-neuron responses in Figs 3c and 4c expressed as normalized activity (color-map plots) were carried out using a moving window of 100-ms slit forward in steps of 20 ms. Furthermore, relative to each neuron peak of activity (equal to 1), we also calculated the burst duration (Figs 3d and 4d) as the time interval between the first bin before and after the peak of activity whose value was higher and lower than (1 − B) × 0.25 + B, respectively, where B is the mean baseline activity. Differences between the burst durations were highlighted by means of a t-test with a significance criterion of p < 0.05. Once we obtained the first bin before the peak of activity whose value was higher than formula previously mentioned, we calculated the neuronal latency as the time interval between the first bin and the onset of the behavioral events of interest. Then, we plotted cumulative distributions of latencies relative to the behavioral events of interest (Figs 3d and 4d). By means of an χ^2^ test performed bin per bin (bin = 100 ms) between cumulative distributions, we established the time intervals in which significant differences in the proportion of neurons were found (χ^2^, p < 0.05).

Population analyses in Supplementary Fig. S2c were carried out comparing trials in which monkeys generated smooth-pursuit eye movements and those in which they generated saccadic eye movements during HRo. Saccades and smooth-pursuit were grouped taking into account for each trial the ocular velocity pattern as shown in Supplementary Fig. S2a.

## Electronic supplementary material

Supplementary Information

## References

[1] Miller EK, Cohen JD (2001). An integrative theory of prefrontal cortex function. Annu Rev Neurosci.

[2] Freedman EG, Sparks DL (1997). Eye-head coordination during head-unrestrained gaze shifts in rhesus monkeys. J Neurophysiol.

[3] Shepherd, S. V. Following gaze: gaze-following behavior as a window into social cognition. *Front Integr Neurosci* **4** (2010). 10.3389/fnint.2010.00005 PMC2859805 20428494

[4] Schall JD (2015). Visuomotor functions in the frontal lobe. Annual Review of Vision Science.

[5] Pierrot-Deseilligny C, Milea D, Muri RM (2004). Eye movement control by the cerebral cortex. Curr Opin Neurol.

[6] Serences JT, Yantis S (2006). Selective visual attention and perceptual coherence. Trends Cogn Sci.

[7] Shepherd SV, Klein JT, Deaner RO, Platt ML (2009). Mirroring of attention by neurons in macaque parietal cortex. Proc Natl Acad Sci USA.

[8] Chapman BB, Pace MA, Cushing SL, B. D (2012). Recruitment of a contralateral head turning synergy by stimulation of monkey supplementary eye fields. J Neurophysiol.

[9] Gandhi NJ, Katnani HA (2011). Motor functions of the superior colliculus. Annu Rev Neurosci.

[10] Knight TA (2012). Contribution of the frontal eye field to gaze shifts in the head-unrestrained rhesus monkey: neuronal activity. Neuroscience.

[11] Knight TA, Fuchs AF (2007). Contribution of the frontal eye field to gaze shifts in the head-unrestrained monkey: effects of microstimulation. J Neurophysiol.

[12] Martinez-Trujillo JC, Wang H, Crawford JD (2003). Electrical stimulation of the supplementary eye fields in the head-free macaque evokes kinematically normal gaze shifts. J Neurophysiol.

[13] Monteon JA, Avillac M, Yan X, Wang H, Crawford JD (2012). Neural mechanisms for predictive head movement strategies during sequential gaze shifts. J Neurophysiol.

[14] Wang Y, Isoda M, Matsuzaka Y, Shima K, Tanji J (2005). Prefrontal cortical cells projecting to the supplementary eye field and presupplementary motor area in the monkey. Neurosci Res.

[15] Yeterian EH, Pandya DN, Tomaiuolo F, Petrides M (2012). The cortical connectivity of the prefrontal cortex in the monkey brain. Cortex.

[16] Ghahremani M, Hutchison RM, Menon RS, Everling S (2016). Frontoparietal Functional Connectivity in the Common Marmoset. Cereb Cortex.

[17] Lanzilotto M, Perciavalle V, Lucchetti C (2015). Orienting movements in area 9 identified by long-train ICMS. Brain Struct Funct.

[18] Gu C, Corneil BD (2014). Transcranial magnetic stimulation of the prefrontal cortex in awake nonhuman primates evokes a polysynaptic neck muscle response that reflects oculomotor activity at the time of stimulation. J Neurosci.

[19] Bristow D, Rees G, Frith CD (2007). Social interaction modifies neural response to gaze shifts. Soc Cogn Affect Neurosci.

[20] Isoda M (2013). Recent advances in social neuroscience research using macaques. Brain Nerve.

[21] Urakawa S, Takamoto K, Ishikawa A, Ono T, Nishijo H (2015). Selective Medial Prefrontal Cortex Responses During Live Mutual Gaze Interactions in Human Infants: An fNIRS Study. Brain Topography.

[22] Schwiedrzik CM, Zarco W, Everling S, Freiwald WA (2015). Face Patch Resting State Networks Link Face Processing to Social Cognition. PLoS Biology.

[23] Bon, L., Lucchetti, C., Portolan, F. & Pagan, M. *MUPRO: a multipurpose robot*. Vol. 112(7) 855–68 (Int J Neurosci, 2002). 10.1080/00207450290025888 12424826

[24] Tsujimoto S, Genovesio A, Wise SP (2010). Evaluating self-generated decisions in frontal pole cortex of monkeys. Nat Neurosci.

[25] Bon, L., Lanzilotto, M. & Lucchetti, C. In *Prefrontal Cortex: Roles*, *Interventions and* Traumas (eds LoGrasso, L. & Morretti, G.) 157–175 (Nova Science Publisher, 2009).

[26] Lanzilotto M, Perciavalle V, Lucchetti C (2013). A new field in monkey’s frontal cortex: premotor ear-eye field (PEEF). Neuroscience & Biobehavioral Reviews.

[27] Lanzilotto M, Perciavalle V, Lucchetti C (2013). Auditory and visual systems organization in Brodmann Area 8 for gaze-shift control: where we do not see, we can hear. Frontiers in behavioral neuroscience.

[28] Bon L, Lucchetti C (1994). Ear and eye representation in the frontal cortex, area 8b, of the macaque monkey: an electrophysiological study. Exp Brain Res.

[29] Bon L, Lucchetti C (2006). Auditory environmental cells and visual fixation effect in area 8B of macaque monkey. Exp Brain Res.

[30] Lanzilotto, M., Perciavalle, V. & Lucchetti, C. Evidence for a functional subdivision of Premotor Ear-Eye Field (Area 8B). *Frontiers in behavioral neuroscience* **8** (2015). 10.3389/fnbeh.2014.00454 PMC4311694 25688190

[31] Lucchetti C, Lanzilotto M, Bon L (2008). Auditory-motor and cognitive aspects in area 8B of macaque monkey’s frontal cortex: a premotor ear-eye field (PEEF). Exp Brain Res.

[32] Bruce CJ, Goldberg ME, Bushnell MC, Stanton GB (1985). Primate frontal eye fields. II. Physiological and anatomical correlates of electrically evoked eye movements. J Neurophysiol.

[33] Chen LL (2006). Head movements evoked by electrical stimulation in the frontal eye field of the monkey: evidence for independent eye and head control. J Neurophysiol.

[34] Elsley JK, Nagy B, Cushing SL, Corneil BD (2007). Widespread presaccadic recruitment of neck muscles by stimulation of the primate frontal eye fields. J Neurophysiol.

[35] Alexander GE, DeLong MR, Strick PL (1986). Parallel organization of functionally segregated circuits linking basal ganglia and cortex. Annu Rev Neurosci.

[36] Sparks DL, Hartwich-Young R (1989). The deep layers of the superior colliculus. Rev Oculomot Res.

[37] Baumann O, Mattingley JB (2010). Medial Parietal Cortex Encodes Perceived Heading Direction in Humans. The Journal of Neuroscience.

[38] Finkelstein A, Las L, Ulanovsky N (2016). 3-D Maps and Compasses in the Brain. Annu Rev Neurosci.

[39] Rubin A, Yartsev MM, Ulanovsky N (2014). Encoding of head direction by hippocampal place cells in bats. J Neurosci.

[40] Sato N, Sakata H, Tanaka YL, Taira M (2006). Navigation-associated medial parietal neurons in monkeys. Proc Natl Acad Sci USA.

[41] Sargolini F (2006). Conjunctive representation of position, direction, and velocity in entorhinal cortex. Science.

[42] Sugihara T, Diltz MD, Averbeck BB, Romanski LM (2006). Integration of auditory and visual communication information in the primate ventrolateral prefrontal cortex. J Neurosci.

[43] Romanski LM, Diehl MM (2011). Neurons responsive to face-view in the primate ventrolateral prefrontal cortex. Neuroscience.

[44] Tsao DY, Schweers N, Moeller S, Freiwald WA (2008). Patches of face-selective cortex in the macaque frontal lobe. Nat Neurosci.

[45] Tian B, Reser D, Durham A, Kustov A, Rauschecker JP (2001). Functional specialization in rhesus monkey auditory cortex. Science.

[46] Recanzone GH (2008). Representation of con-specific vocalizations in the core and belt areas of the auditory cortex in the alert macaque monkey. J Neurosci.

[47] Perrett DI (1985). Visual cells in the temporal cortex sensitive to face view and gaze direction. Proc R Soc Lond B Biol Sci.

[48] Marciniak K, Atabaki A, Dicke PW, Thier P (2014). Disparate substrates for head gaze following and face perception in the monkey superior temporal sulcus. eLife.

[49] Caruana F, Jezzini A, Sbriscia-Fioretti B, Rizzolatti G, Gallese V (2011). Emotional and social behaviors elicited by electrical stimulation of the insula in the macaque monkey. Curr Biol.

[50] Jezzini, A. *et al*. A shared neural network for emotional expression and perception: an anatomical study in the macaque monkey. *Front Behav Neurosci* **9** (2015). 10.3389/fnbeh.2015.00243 PMC4585325 26441573

[51] Jezzini A, Caruana F, Stoianov I, Gallese V, Rizzolatti G (2012). Functional organization of the insula and inner perisylvian regions. Proc Natl Acad Sci USA.

[52] Hoffman KL, Gothard KM, Schmid MC, Logothetis NK (2007). Facial-expression and gaze-selective responses in the monkey amygdala. Curr Biol.

[53] Bullock, K. R., Pieper, F., Sachs, A. J. & Martinez-Trujillo, J. C. Visual and presaccadic activity in area 8Ar of the macaque monkey lateral prefrontal cortex. *J Neurophysiol* **15** (2017). 10.1152/jn.00278.2016 PMC5494359 28298302

[54] Duffy CJ (1998). MST neurons respond to optic flow and translational movement. J Neurophysiol.

[55] Fukushima J, Akao T, Kurkin S, Kaneko CR, Fukushima K (2006). The vestibular-related frontal cortex and its role in smooth-pursuit eye movements and vestibular-pursuit interactions. J Vestib Res.

[56] Fukushima, K., Akao, T., Kurkin, S. & Fukushima, J. Role of vestibular signals in the caudal part of the frontal eye fields in pursuit eye movements in three-dimensional space. *Ann N Y Acad Sci*, 272–282 (2005). 10.1196/annals.1325.026 15826981

[57] Gu Y, Cheng Z, Yang L, DeAngelis GC, Angelaki DE (2016). Multisensory Convergence of Visual and Vestibular Heading Cues in the Pursuit Area of the Frontal Eye Field. Cereb Cortex.

[58] Takahashi K (2007). Multimodal coding of three-dimensional rotation and translation in area MSTd: comparison of visual and vestibular selectivity. Journal of Neuroscience.

[59] Oemisch M, Westendorff S, Everling S, Womelsdorf T (2015). Interareal Spike-Train Correlations of Anterior Cingulate and Dorsal Prefrontal Cortex during Attention Shifts. J Neurosci.

[60] Caruana F (2014). Human cortical activity evoked by gaze shift observation: an intracranial EEG study. Hum Brain Mapp.

[61] Shmuelof, L. & Zohary, E. Mirror-image representation of action in the anterior parietal cortex. *Nat Neurosci* **11**, 1267-1269, doi:http://www.nature.com/neuro/journal/v11/n11/suppinfo/nn.2196_S1.html (2008). 10.1038/nn.2196 18820694

[62] di Pellegrino G, Fadiga L, Fogassi L, Gallese V, Rizzolatti G (1992). Understanding motor events: a neurophysiological study. Exp Brain Res.

[63] Rizzolatti G, Craighero L (2004). The mirror-neuron system. Annu Rev Neurosci.

[64] Gallese V, Fadiga L, Fogassi L, Rizzolatti G (1996). Action recognition in the premotor cortex. Brain.

[65] Rizzolatti G, Fadiga L, Gallese V, Fogassi L (1996). Premotor cortex and the recognition of motor actions. Brain Res Cogn Brain Res.

[66] Bonini L (2016). The Extended Mirror Neuron Network: Anatomy, Origin, and Functions. Neuroscientist.

[67] Bonini L, Ferrari PF (2011). Evolution of mirror systems: a simple mechanism for complex cognitive functions. Annals of the New York Academy of Sciences.

[68] Bonini L, Maranesi M, Livi A, Fogassi L, Rizzolatti G (2014). Space-Dependent Representation of Objects and Other’s Action in Monkey Ventral Premotor Grasping Neurons. The Journal of Neuroscience.

[69] Pani P, Theys T, Romero MC, Janssen P (2014). Grasping Execution and Grasping Observation Activity of Single Neurons in the Macaque Anterior Intraparietal Area. Journal of Cognitive Neuroscience.

[70] Chapman BB, Corneil BD (2014). Short-duration stimulation of the supplementary eye fields perturbs anti-saccade performance while potentiating contralateral head orienting. Eur J Neurosci.

[71] Ghazanfar AA, Chandrasekaran C, Morrill RJ (2010). Dynamic, rhythmic facial expressions and the superior temporal sulcus of macaque monkeys: implications for the evolution of audiovisual speech. Eur J Neurosci.

[72] Romanski LM, Goldman-Rakic PS (2002). An auditory domain in primate prefrontal cortex. Nat Neurosci.

[73] Romanski LM, Averbeck BB, Diltz M (2005). Neural representation of vocalizations in the primate ventrolateral prefrontal cortex. J Neurophysiol.

[74] Roy, A., Shepherd, S. V. & Platt, M. L. Reversible inactivation of pSTS suppresses social gaze following in the macaque (Macaca mulatta). *Social cognitive and affective neuroscience*, nss123 (2012). 10.1093/scan/nss123 PMC3907927 23171617

[75] Lucchetti, C., Lanzilotto, M., Perciavalle, V. & Bon, L. Neuronal activity reflecting progression of trials in the pre-supplementary motor area of macaque monkey: An expression of neuronal flexibility. *Neuroscience Letters* **506**, 33–38, doi:10.1016/j.neulet.2011.10.043 (2012). 10.1016/j.neulet.2011.10.043 22040673

[76] Shepherd SV, Lanzilotto M, Ghazanfar AA (2012). Facial muscle coordination in monkeys during rhythmic facial expressions and ingestive movements. Journal of Neuroscience.

