# Supplementary material for: Neuronal Encoding of Self and Others’ Head Rotation in the Macaque Dorsal Prefrontal Cortex
Source: Sci Rep. 2017 Aug 17;7:8571. doi: 10.1038/s41598-017-08936-5 (PMC5561028; doi:10.1038/s41598-017-08936-5)
Supplement: Supplementary file 1 — Supplementary Information [file 41598_2017_8936_MOESM1_ESM.pdf]

Neuronal Encoding of Self and Others' Head Rotation in the Macaque Dorsal Prefrontal Cortex

Lanzilotto M, Gerbella M, Perciavalle V, Lucchetti C

Supplementary Information (SI)

Anatomical injections and histological reconstruction

In MK3 and MK4, neural tracers were injected in the same sectors of BA 9/46dr investigated in the electrophysiological study carried out in MK1 and MK2. To this purpose, the functional maps of MK1 and MK2 were overlapped on the anatomical reconstruction of their brains (see below for details) and then superimposed on MRI scans of MK3 and MK4 brains. Then, under general anesthesia (ketamine hydrochloride, 5 mg/kg i.m. and medetomidine hydrochloride, 0.1 mg/kg i.m.) and aseptic conditions, each animal was placed in a stereotaxic apparatus and an incision was made in the scalp. The skull was trephined to remove the bone overlying the BA 9/46dr, and the dura was opened. The neural tracers lucifer yellow (10000 MW, Lucifer Yellow Dextran, LYD, 10% in 0.1 M phosphate buffer, pH 7.4; Invitrogen-Molecular Probes) and wheat germ agglutinin-horseradish (WGA, 4% in distilled water, Sigma, St. Louis, MO, USA) were slowly pressure-injected through a glass micropipette (tip diameter: 50–100 µm) attached to a 1- or 10-µL Hamilton microsyringe (Reno, NV, USA) at 1.2–1.5 mm below the cortical surface. After the tracer injections were completed, the dural flap was sutured, the bone was replaced, and the superficial tissues were sutured in layers. During surgery, hydration was provided with saline, and the temperature was maintained using a heating pad. Heart rate, blood pressure, respiratory depth, and body temperature were continuously monitored. Upon recovery from anesthesia, the animals were returned to their home cages and closely monitored. Dexamethasone and prophylactic broad-spectrum antibiotics were administered pre- and postoperatively. Furthermore, analgesics were administered intra- and postoperatively. After appropriate survival periods for the injected monkeys (MK3, 28 days for LYD; MK4, 2 days for WGA) and at the end of the physiological experiments for the recorded monkeys (MK1 and MK2), each animal was anaesthetized with ketamine hydrochloride (15 mg/kg i.m.) followed by an intravenous lethal injection of pentobarbital sodium and perfused with saline, 3.5-4% paraformaldehyde, and 5% glycerol, prepared in 0.1M phosphate buffer, pH 7.4, through the left cardiac ventricle. About one week before sacrificing the recorded monkeys (MK1 and MK2), marking lesions (D.C., 10 µA, 15 s) were made in the recorded area, respectively medially (~ 2 mm) and laterally (~ 8 mm) in respect to the interhemispheric line. For all monkeys, the brain was then blocked coronally on a stereotaxic apparatus, removed from the skull, photographed, and placed in 10% buffered glycerol for 3 days and 20% buffered glycerol for 4 days. Finally, each brain was cut frozen into coronal sections of 60-µm thickness. For all monkeys, each second and fifth section of a series of five were stained using the Nissl method (thionin, 0.1% in 0.1 M acetate buffer, pH 3.7). In MK1 and MK2, the locations of the electrode tracks were assessed under an optical microscope in Nissl-stained sections, and then plotted and digitalized together with the outer and inner borders of the cerebral cortex using a computer-based charting system (for details of the procedure see [70]). To obtain 3-D volumetric monkey-brain reconstructions, which contained the data concerning the location of the electrode traces and of the electrolytic lesions, the data from individual sections were also imported into custom-made software. Finally, the schematic drawing of the recorded neurons was superimposed, based on the position of the electrolytic lesions, on a dorsal view of both monkey-brain 3-D reconstructions. In MK3 and MK4, one series of each fifth section was processed for the visualization of LYD and WGA, respectively. Specifically, MK3 sections were incubated for 72 h at 4° C in a primary antibody solution of rabbit anti-LYD (1:3,000; Invitrogen), 0.3% Triton, and 5% normal goat serum in 0.01 M phosphate-buffered saline at pH 7.4, and for 1 h in biotinylated secondary antibody (1:200, Vector), 0.3% Triton, and 5% normal goat serum in 0.01 M phosphate-buffered saline at pH 7.4. MK4 sections were incubated overnight at room temperature in a primary antibody solution of goat anti-WGA (1:3,000; Invitrogen), 0.3% Triton, and 5% normal horse serum in 0.01 M phosphate-buffered saline at pH 7.4, and for 1 h in biotinylated secondary antibody (1:200, Vector), 0.3% Triton, and 5% normal horse serum in 0.01 M phosphate-buffered saline at pH 7.4. Finally, LYD and WGA labeling was visualized using Vector SG peroxidase substrate kit (SK-4700, Vector) and the Vectastain ABC kit (Vector Laboratories) and 3,3'- diaminobenzidine (DAB) as a chromogen, respectively. For the WGA, the reaction product was intensified with cobalt chloride and nickel ammonium sulfate. The

distribution of retrograde (for both tracer injections) and anterograde (for LYD injections) labeling was analyzed in sections every 600 µm for the cortex and in sections every 300 µm for the subcortical structures, using a computer-based charting system. Data from individual sections were also imported into the aforementioned 3-D reconstruction software. The criteria and maps used for the areal attribution of the labeling were based on Paxinos Atlas.

Supplementary Figures

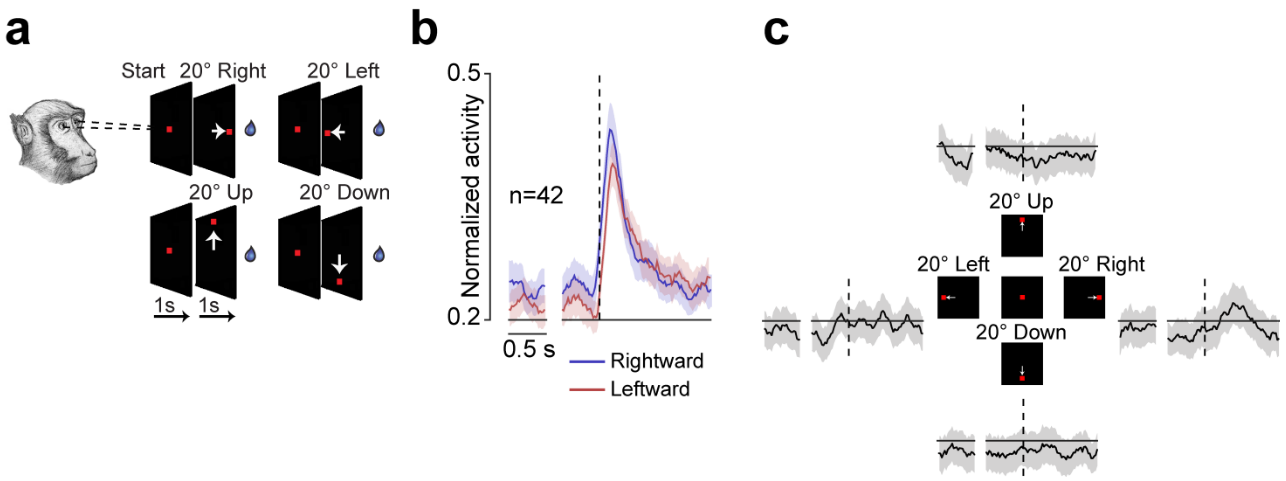

Figure S1 Population activity of head-rotation neurons tested during the HRe (**b**) and ST (**c**). (**a**) Schematic representation of the visually guided saccade task (ST). (**b**) Population activity of 42 head-rotation neurons during rightward and leftward head rotation in the HRe. Conventions as in Fig. 3. (**c**) Population activity of the same 42 neurons shown in (**b**) aligned to the onset of 20° saccadic eye movement directed leftward, upward, rightward, and downward. Epoch immediately before the dashed line represents the neuronal activity during the premovement period and, before the gap, neuronal activity during the baseline period, when the monkey was fixating in the central position. The gray shaded area around each curve represents 1 standard error around the averaged population response. ANOVA (see Materials and Methods for details) showed no significant effect during ST ($F = 1.24$, $p = 0.27$). To enable a direct comparison between population responses, scales of x- and y-axes are the same for (**b**) and (**c**).

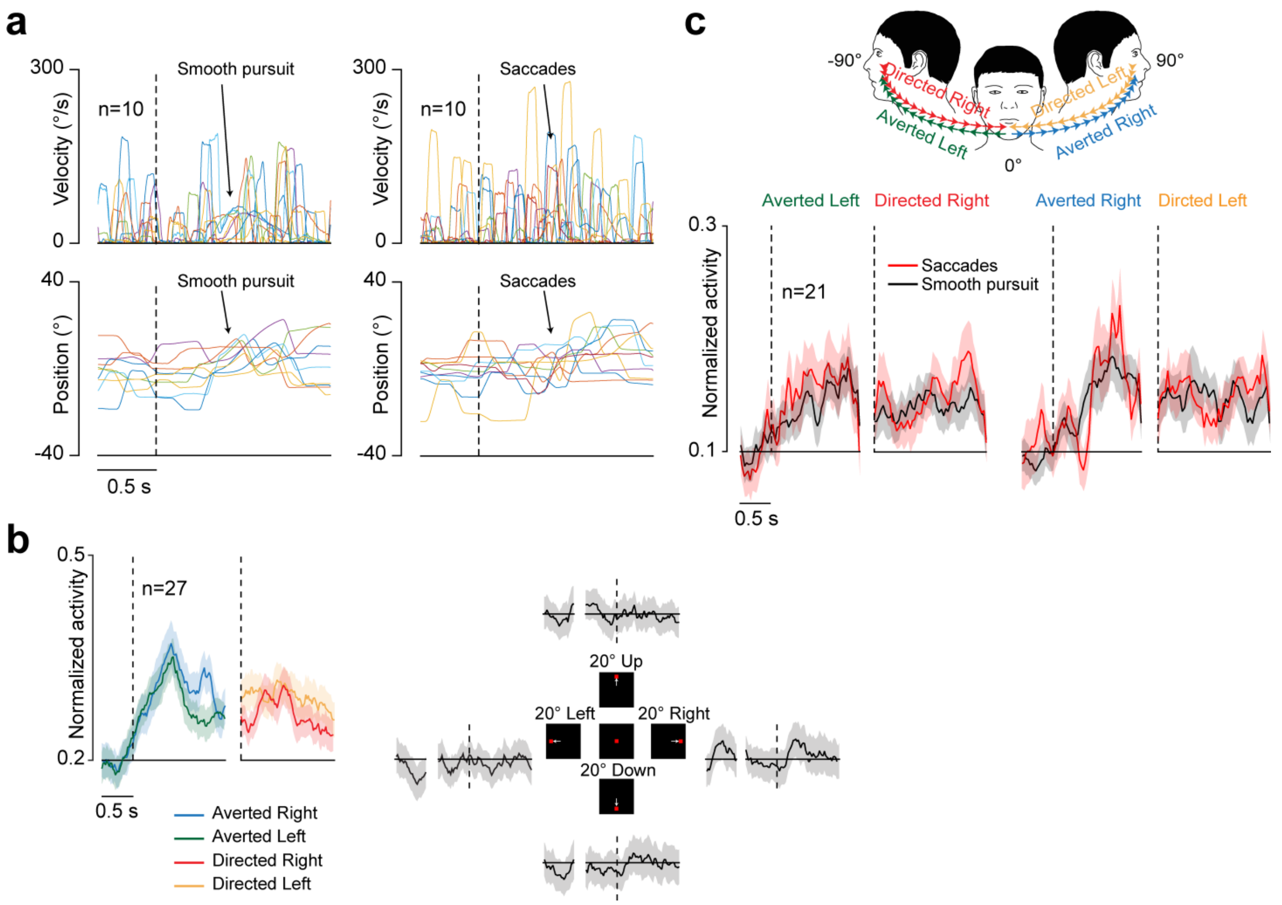

Figure S2 Population activity of visually triggered neurons tested during the HRo and ST. (**a**) Left, plots show velocity profile (upper) and position (bottom) of 10 randomly sampled trials of smooth-pursuit eye movements (left) and saccades (right) recorded during the HRo. Each color represents one trial. (**b**) Left, population activity of 27 visually triggered neurons aligned (dashed lines) to the epochs of the experimenter's head rotation. Conventions as in Fig. 4. By means of 2 x 3 repeated measures ANOVA (factors: Hemifield and Epoch) applied to visually triggered neurons, we identified a significant neuronal response during all tested epochs ($F = 31.33$, $p < 0.001$) without directional preference. Right, population activity of the same 27 neurons aligned to the onset of 20° saccadic eye movements directed leftward, upward, rightward, and downward. Conventions as in Fig. S1. The gray shaded area around each curve represents 1 standard error around the averaged population response. To enable a direct comparison between population responses, scales of x- and y-axes are the same for both neuronal populations left and right. By means of a 4 x 4 repeated measures ANOVA (factors: Direction and Epoch), we observed that neuronal population was not modulated during any epoch or direction of ST ($F = 0.84$, $p = 0.58$). (**c**) Upper, drawing of the experimenter's head rotation during the HRo. Bottom, population activity of 21 visually triggered neurons, distinguishing between trials in which monkeys generated smooth-pursuit eye movements (black) and those in which they generated saccadic eye movements (red). Conventions as in Fig. 4. By means of 2 x 3 repeated measures ANOVAs (factors: Hemifield and Epoch) applied to each tested hemifield, we identified a significant neuronal response during all averted and directed epochs (left hemifield, $F = 7.59$, $p < 0.001$; right hemifield, $F = 10.99$, $p < 0.001$), but no significant difference was found in the neuronal activity associated with monkeys' oculomotor behavior (saccades vs. smooth pursuit) (left hemifield, $F = 1.03$, $p = 0.37$; right hemifield, $F = 0.19$, $p = 0.83$).

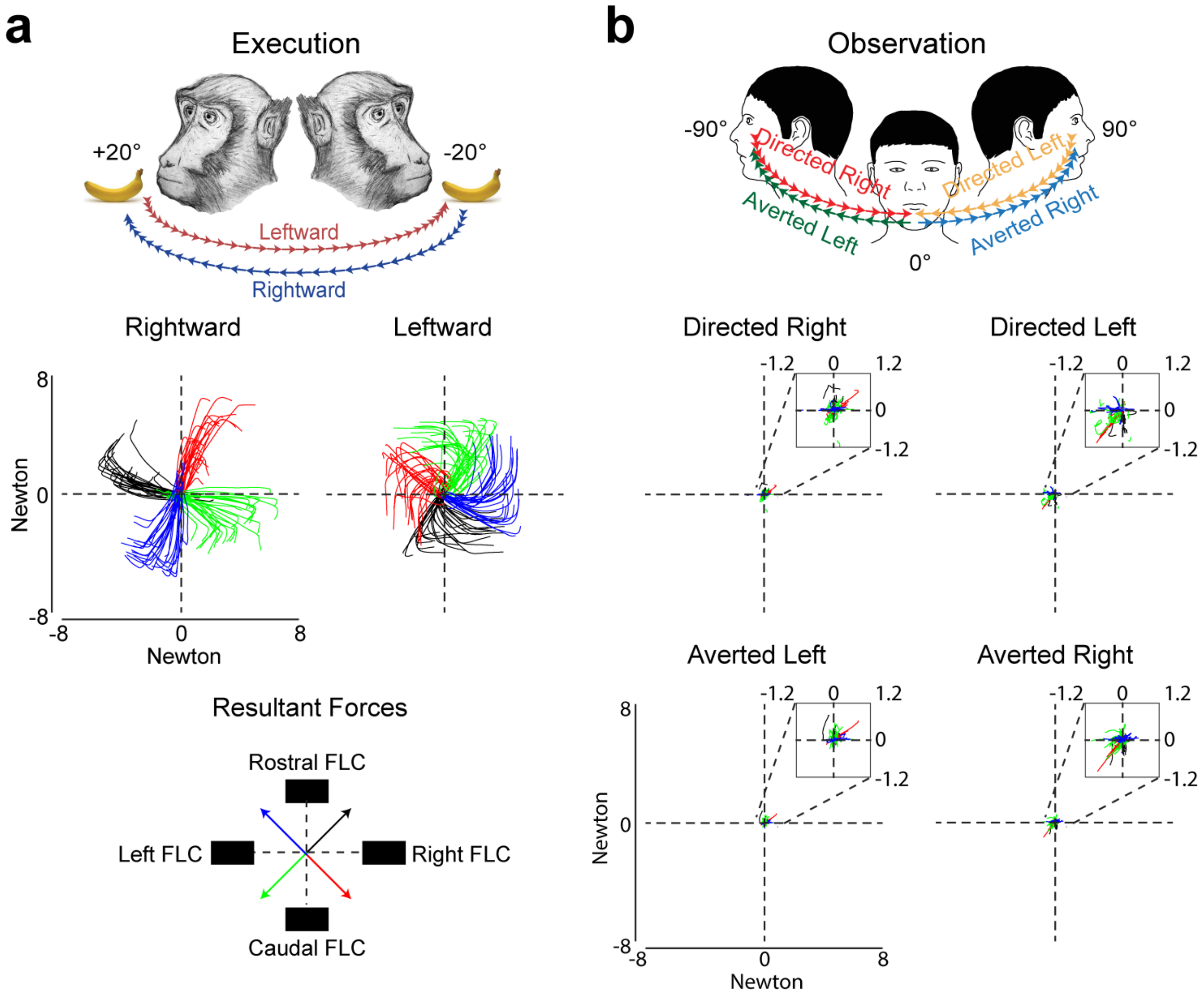

Figure S3 Monkey's neck forces exerted during both the HRe (**a**) and HRo (**b**), detected during the simultaneous recording of visually triggered neurons. (**a**) Plots represent the neck forces applied by the monkey during active rightward and leftward head rotation. Each line describes the average of resultant forces of at least 10 trials between two consecutive flexion load cells (FLCs), as shown by drawing, bottom. See Materials and Methods for details regarding MUPRO. Positive values indicate that the FLC is charging up and that the monkey is then applying force in that direction. Negative values indicate that the FLC is discharging and that monkey is then applying force in the opposite direction. Zero indicates the equilibrium point. During rightward head rotation, for example, in the first phase of the movement, monkeys apply forces mainly toward the caudal and left FLCs to win the inertia of MUPRO, whereas in the final phase of the rotation movement, they apply forces mainly toward the rostral and right FLCs. (**b**) Plots represent the monkey's neck forces recorded during each epoch of the observation task. Plots in the squares rescale the neck forces, which are clearly negligible relative to the execution condition. Other conventions as above. It is clear that during the observation task, monkeys did not apply isometric neck forces in order to attempt to rotate the head, which could explain the enhancement of the neuronal response of visually triggered neurons.

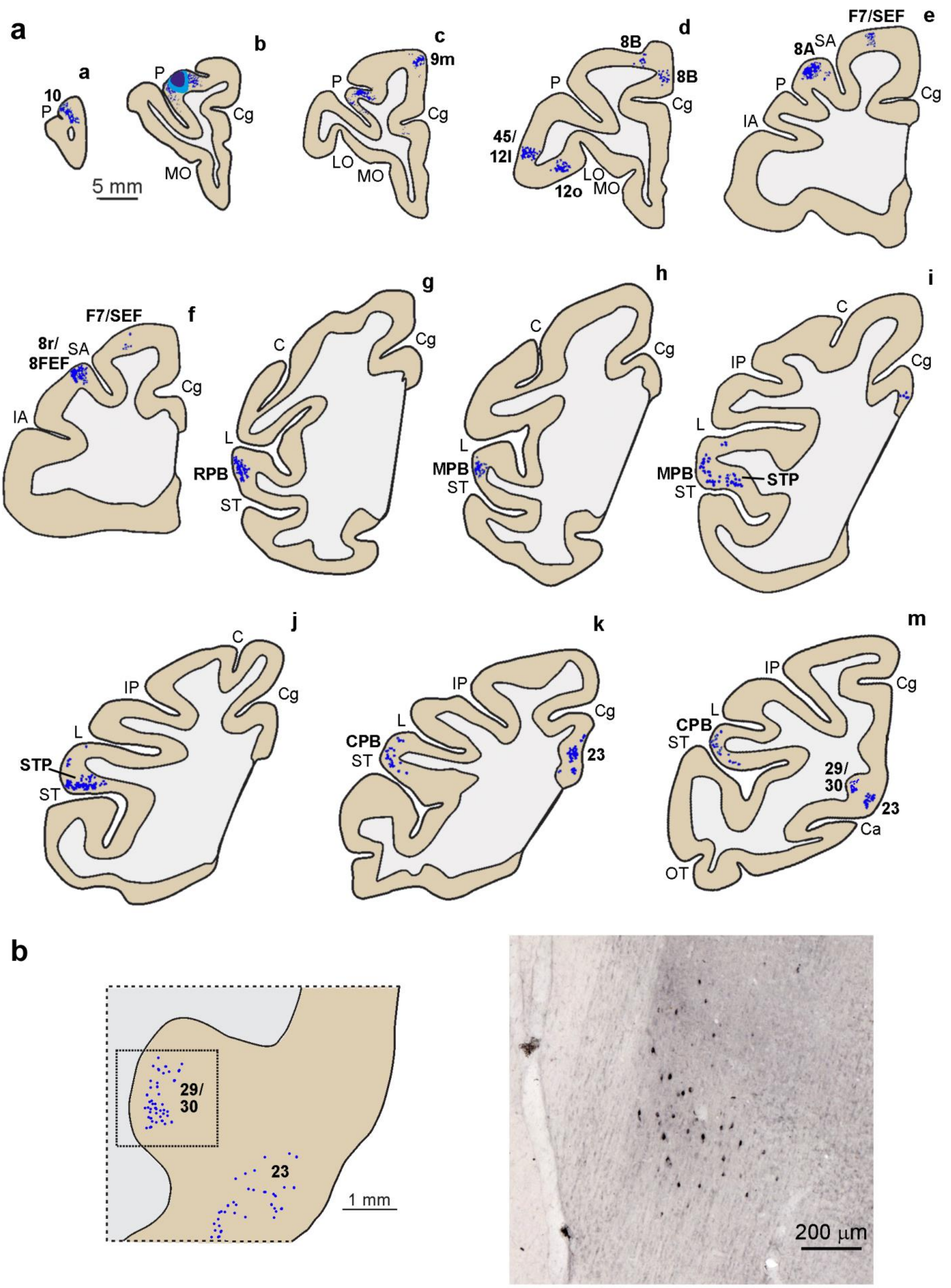

Figure S4 Distribution of the retrograde (blue dots) labeling observed following WGA injection in MK4, shown in drawings of coronal sections (**a**) arranged in a rostral-to-caudal order (a–m). Each blue dot corresponds to one labeled cell. (**b**) Drawing, left, and photomicrograph, right, of the retrosplenial cortex. Abbreviations as in Figures 2 and 6.
